# Efficacy of Bravecto^®^ Plus spot-on solution for cats (280 mg/ml fluralaner and 14 mg/ml moxidectin) in the prevention of feline *Aelurostrongylus abstrusus* infection evaluated in a multi-diagnostic approach

**DOI:** 10.1186/s13071-024-06270-0

**Published:** 2024-04-24

**Authors:** Marie-Kristin Raulf, Katharina Raue, Nadja Rohdich, Eva Zschiesche, Jonathan Raue, Kristina Merhof, Holger A. Volk, Manuela Schnyder, Simone Morelli, Donato Traversa, Rainer K. A. Roepke, Christina Strube

**Affiliations:** 1 Institute for Parasitology, Centre for Infection Medicine, University of Veterinary Medicine Hannover, Buenteweg 17, 30559 Hanover, Germany; 2 MSD Animal Health Innovation GmbH, Schwabenheim, Germany; 3 Department for Small Animal Medicine and Surgery, University of Veterinary Medicine Hannover, 30559 Hannover, Germany; 4 Institute of Parasitology, Vetsuisse Faculty, University of Zurich, 8057 Zurich, Switzerland; 5 Department of Veterinary Medicine, University of Teramo, Teramo, Italy

**Keywords:** Aelurostrongylosis, Feline lungworms, Macrocylic lactones, Moxidectin, Treatment, Prevention, Control

## Abstract

**Background:**

*Aelurostrongylus abstrusus* is one of the most important respiratory nematodes of felines. Infections may lead to respiratory clinical signs with varying severity or even death, emphasizing the need for preventive treatment of cats with outdoor access to circumvent patent infections.

**Methods:**

Therefore, the preventive efficacy of a spot-on formulation of 280 mg/ml fluralaner and 14 mg/ml moxidectin (Bravecto^®^ Plus spot-on solution for cats, MSD) against *A. abstrusus* was evaluated in a negative controlled, randomized and partially blinded efficacy study with 28 purpose-bred cats in a non-terminal design. In three different treatment regimes, the minimum recommended dose of 40 mg fluralaner and 2.0 mg moxidectin/kg bodyweight (BW) was administered once at 12, 8 or 4 weeks (study group G1, G2 and G3, respectively) prior to experimental infection with 300 third-stage *A. abstrusus* larvae, while G4 served as placebo-treated control.

**Results:**

From 30 to 46 days post infection (dpi; SD 114 to 130), faeces were sampled to monitor first-stage larvae (L1) excretion for efficacy determination. Secondary efficacy criteria, including respiratory parameters, serological antibody levels and computed tomography (CT) findings, were assessed once before enrolment (SD −7 to −1) and before infection (SD 75 to 83). After infection, CT evaluation was performed once at 47–50 dpi (SD 131 to 134), and respiratory parameters and antibody levels were regularly assessed twice or once a week, respectively (1 up to 78 dpi, SD 85 up to 162). All animals in the control group excreted L1 by 33–37 dpi and remained positive throughout the study period from 41 to 46 dpi (SD 125 to 130). In the treatment groups, only one animal each of G1 and G2 excreted L1 at two consecutive days, and four cats of G1, two of G2 and three of G3 were positive on single occasions. While the geometric mean (GM) of the maximum number of excreted L1 per 5 g of faeces was 7380.89 in the control group (G4), GMs were significantly lower in the treatment groups with 1.63 in G1, 1.37 in G2 and 0.79 in G3. Thus, based on GMs, the reduction in excreted L1 exceeded 99.9% in all three treatment groups. Based on CT severity scores, all lungs of the animals of the control group showed severe pulmonary changes post infection, whereas lungs of the cats of the treatment groups were either unaltered (4 animals), mildly (11 animals), or moderately altered (5 animals). Moreover, seroconversion was observed in all cats of the control group, but not in those of the treatment groups.

**Conclusions:**

The combination of diagnostic methods used in this non-terminal study yielded coherent and reliable results. A single administration of Bravecto^®^ Plus spot-on solution for cats was well tolerated and effective in the prevention of aelurostrongylosis for at least 12 weeks.

**Graphical Abstract:**

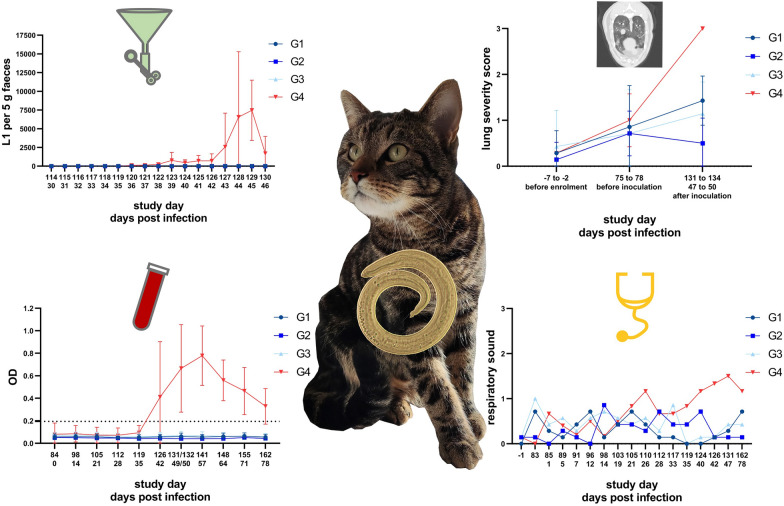

**Supplementary Information:**

The online version contains supplementary material available at 10.1186/s13071-024-06270-0.

## Background

The metastrongylid *Aelurostrongylus abstrusus*, often referred to as the “cat lungworm”, is one of the most important respiratory nematodes of domestic cats occurring throughout the world [1–3]. In domestic cats of Europe, *A. abstrusus* is widespread with prevalence ranging from 0.1% to 13.6% in Northern and Central Europe [4–12], and from 7.7% to 27.5% in Eastern and Southern Europe [3, 13–16]. Adult worms inhabit the alveoli, alveolar ducts and terminal bronchioles in the feline lung parenchyma, inducing respiratory alterations of varying severity. Clinical signs may range from sneezing and nasal discharge to coughing, dyspnoea and tachypnoea up to respiratory failure and even death [17–20]. However, many infected cats show subclinical signs or present with mild to moderate respiratory disease [1]. Even when cats do not show noticeable respiratory signs, pathological alterations in the lungs are frequently detected [19, 21]. Thus, image acquisition methods such as radiographs or computed tomography (CT) are helpful supporting tools for diagnosis of aelurostrongylosis [22, 23]. Lung changes can be observed over the entire course of infection, spanning from prepatency, where lung damage has most likely already occurred [21], to patency and even postpatency, where pathological alterations may be visible for up to several weeks or months [21, 22]. Low and high infectious doses were associated with the severity of the pulmonary lesions, which changed during the course of disease [23]. However, since alterations in lung pathology may resemble those of other respiratory diseases (e.g. feline asthma), additional examination methods need to be implemented to achieve definitive diagnosis [22, 24, 25]. The most common methods used for diagnosis of *A. abstrusus* involve the detection of first-stage larvae (L1) in faeces via the Baermann method, of larval and adult DNA in faeces and pharyngeal swaps via PCR and of anti-*A. abstrusus* major sperm protein (MSP) antibodies in serum via ELISA using bovine lungworm MSP as a diagnostic antigen [26–28].

Owing to the widespread occurrence and clinical relevance of *A. abstrusus*, regular copromicroscopic examinations or anthelmintic treatment of cats with outdoor access should be encouraged. In Europe, several products are approved to control aelurostrongylosis in cats. These products contain different formulations of moxidectin, eprinomectin, emodepside and fenbendazole; however, not all these active substances (particularly fenbendazole, only nationally registered in the UK) are actually licensed or marketed against feline aelurostrongylosis in the individual countries. Some of these products are only suitable for the treatment of a pre-existing infection and need to be applied more than once to induce an efficient parasiticidal effect. While this is true for formulations containing emodepside, which are only licensed against adult *A.* *abstrusus* and have to be administered twice at a 2-week interval [29, 30], both eprinomectin and moxidectin hold the potential for preventive efficacy. As lung damage manifests during prepatency [21], preventive efficacy is beneficial. Currently, eprinomectin is used in licensed products, being able to prevent clinical aelurostrongylosis and larval excretion by acting against third- (L3) and fourth-stage (L4) larvae as well as adults of *A.* *abstrusus* [31, 32]. A spot-on preparation containing moxidectin 1% was effective in preventing pulmonary damage and patent *A. abstrusus* infections when administered at the minimum treatment dose of 1 mg/kg bodyweight (BW) once before and once after infection at 4-week intervals, while three applications at 4-week intervals were required for effective treatment of a pre-existing patent infection [33]. While the above-mentioned formulations containing moxidectin or eprinomectin must be administered monthly for effective prevention of aelurostrongylosis, a recent study demonstrated that a single dose of a spot-on formulation containing 14 mg/ml moxidectin, administered at the minimum recommended dose of 2 mg/kg BW, reliably prevented aelurostrongylosis over a period of at least 12 weeks [34]. To confirm these findings, the present study was conducted to evaluate the use of a combination of moxidectin and fluralaner for the prevention of clinical aelurostrongylosis in cats as required by the European Medicines Agency (EMA) for the approval of a veterinary medicinal product. While in the past, a non-terminal efficacy study using only larval counts was done for an eprinomectin-containing product [32], this study marks the first attempt using the 3R principle to establish an alternative non-terminal study design for the investigation of preventative efficacy of actives against *A. abstrusus* in cats. The objective of this study was to evaluate efficacy using a combination of different diagnostic methods with the goal of abolishing the use of terminal laboratory efficacy studies as previously proposed by Traversa and Joachim [35] for demonstrating the prevention of feline aelurostrongylosis, which are currently required per VICH guideline 7 (VICH, 2022a).

## Methods

### Study design

**Fig. 1:**
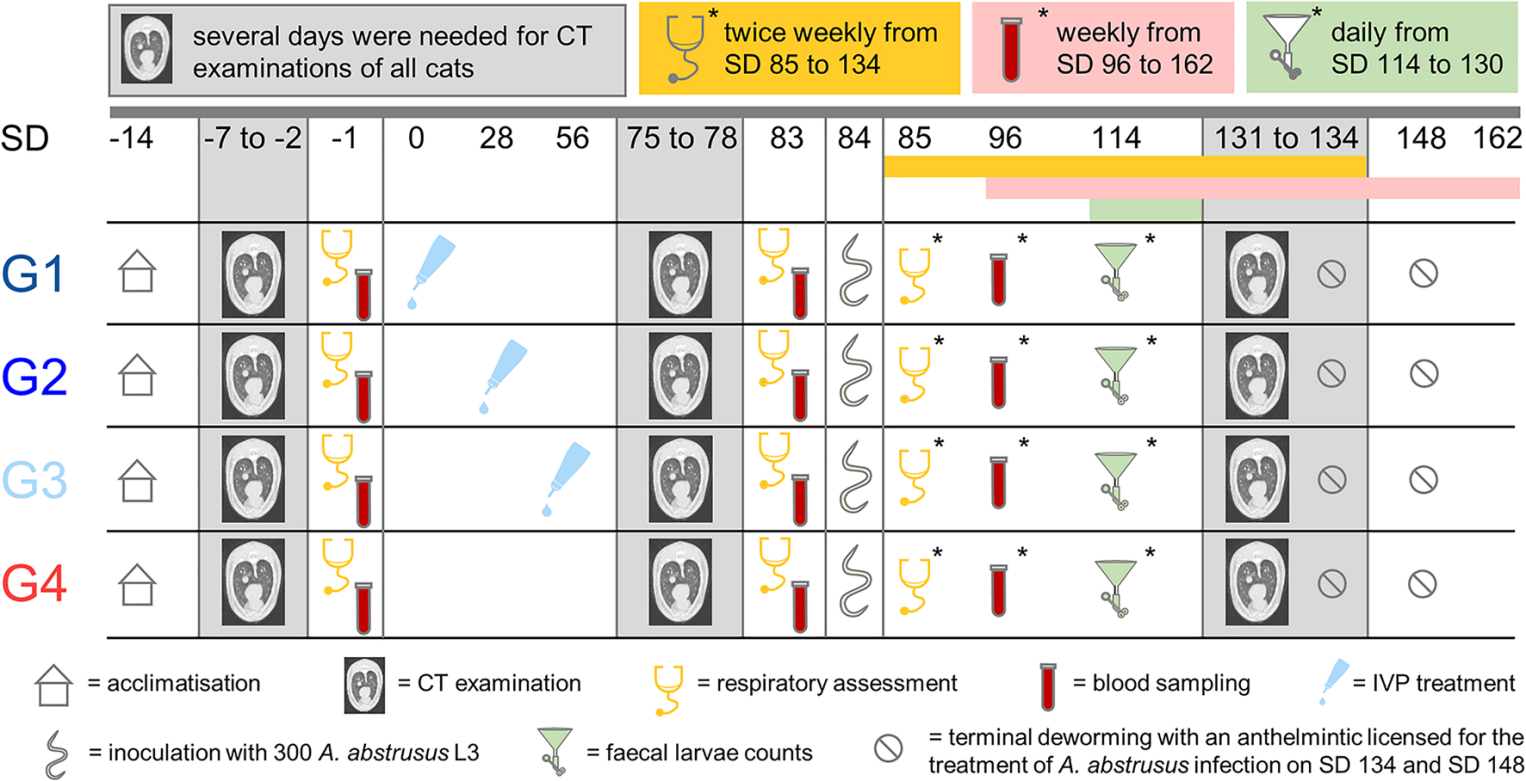
Overview on the design of the confirmation study to assess the preventive efficacy of Bravecto^®^ Plus spot-on solution (IVP, minimum recommended dose of 40 mg fluralaner and 2.0 mg moxidectin/kg body weight; G1, G2, G3). The control group (G4) received a placebo (0.9% saline solution), as did the treatment groups on the study days when they did not receive IVP treatment. CT: computed tomography; L3: third-stage larvae; SD: study day

The preventive efficacy of 2.0 mg moxidectin and 40 mg fluralaner/kg BW against aelurostrongylosis in cats was evaluated in a negative-controlled, randomized and partially blinded efficacy study (second dose confirmation study), in which the investigational veterinary product (IVP, Bravecto^®^ Plus spot-on solution for cats) was applied topically in a single dose 12, 8 or 4 weeks prior to experimental *A. abstrusus* infection. The study was performed in accordance with VICH guideline 9 “Good Clinical Practice” [36]. Deviating from VICH guideline 7 “Efficacy of anthelmintics: General requirements” [37], a combination of diagnostic methods (i.e., faecal larval counts, respiratory assessments, CT imaging and serology) at specific time points based on the parasite’s life cycle and the features of aelurostrongylosis was used instead of worm counts at necropsy. A detailed study design is given in Fig. 1.

### Study animals

Twenty-eight purpose-bred female (*n* = 10, intact) and male (*n* = 18, neutered) European Shorthair cats at the age of 6–7 months (mean 6.8 months) with BW between 2.2 and 4.4 kg (mean 3.4 kg) were obtained from a commercial breeder. All cats were acclimatized to the husbandry conditions at the Institute for Parasitology, University of Veterinary Medicine Hannover, Germany, for at least 7 days prior to first assessments. For inclusion in the study, the cats had to be clinically healthy, free of clinical and CT findings indicative of a prior lungworm infection,  ≥ 9 weeks of age, not treated with drugs interfering with the establishment of the experimental infection, having a BW ≥ 1.2 kg on study day (SD) 0, and at least two faecal samples negative for nematode eggs or larvae during the acclimatization phase.

The study animals were kept indoors in environmentally controlled rooms in groups of two or three cats within their study groups. The rooms were equipped with shelves and suspended pipes for jumping, climbing and observing, cardboard boxes as hiding places, scratch poles, toys and litter pans. Animals had a view of pasture grazed by donkeys and sheep. Upon arrival, after treatment, after experimental infection and for faecal sample collection, cats were separated by dividing the pen with acrylic glass, to prevent physical but allow audio-visual and olfactory contact with other animals. The animals received an age-appropriate commercial cat diet at the recommended rates and water ad libitum. Cats were trained with treats for handling procedures. After completion of the study, cats were adopted by private owners.

### Group allocation and treatment regimes

Prior to acclimatization, all 28 cats were ranked by sex and descending BW and were randomly allocated to the four study groups via a randomization table. Detailed information on the homogeneity of study groups according to age, weight and sex is listed in Additional file 1: Table S1.

The cats in experimental groups G1, G2 and G3 were treated once with Bravecto^®^ Plus spot-on solution for cats at the minimum recommended dose of 40 mg fluralaner and 2.0 mg moxidectin/kg BW on SD 0, SD 28 and SD 56, respectively (Fig. 1). G4 served as a control and received a placebo (0.9% saline solution) on the above-mentioned study days. To ensure blinding, groups that were not dosed with the IVP on the respective treatment days received the placebo as well, i.e. G1 on SD 28 and 56, G2 on SD 0 and 56, and G3 on SD 0 and 28. Administration sites of all cats were examined for any abnormal signs at 10 min and at 24 h ± 4 h after treatment and at the end of the efficacy evaluation at 47–50 days post infection (dpi) (SD 131 to 134). On SD 134 and 148, animals were treated with an anthelmintic (Profender^®^) licensed for the treatment of *A. abstrusus* infection to release them from the study.

### Health monitoring

Qualified and trained animal caretakers performed general health observations once a day. Abnormalities were reported to a veterinarian with concomitant comprehensive veterinary examination of the affected animal, if considered necessary. Physical examinations were performed regularly, i.e. before treatment, before infection, before CT imaging and at the end of the study.

### Preparation of *Aelurostrongylus abstrusus* L3 and experimental inoculation

Infection of land snails (*Cornu aspersum*) with 500 *A. abstrusus* L1 obtained from faeces of naturally infected cats and maintenance of snails until development of L3 were performed at the Department of Veterinary Medicine, University of Teramo, Italy, as previously described [38]. Absence of natural metastrongylid infections was confirmed by morphological and microscopical examination of 10% of the snails before the start of breeding [38, 39].

On the day of experimental inoculation of the study cats (SD 84), infective L3 were retrieved from the muscular foot of the snails using an established protocol [40]. Briefly, 10–15 feet were minced and digested in 100–250 ml of a pepsin-HCl solution (6 g pepsin [≥ 2000 FIP-U/g, Carl Roth GmbH, Karlsruhe, Germany] and 7 ml HCl 37% [Carl Roth GmbH, Karlsruhe, Germany] in 1 L water) at 41 °C for 30 min while stirring. Digested material was sieved and centrifuged at 600 × *g* for 5 min, and obtained sediment was washed with tap water followed by another centrifugation step. The arithmetic mean number of L3 in five aliquots of 100 µl sediment was determined to calculate the volume needed for an inoculation dose of approximately 300 L3. Until use, L3 were kept at 20–25 °C.

For inoculation, cats were anaesthetized by intramuscular injection of medetomidine (80 µg/kg BW, Domitor^®^, Vetoquinol, Ismaning, Germany) and ketamine (7.5 mg/kg BW, Ketamin^®^ 10%, bela-pharm, Vechta, Germany). To avoid regurgitation of the inoculum, metoclopramide (0.3 mg/kg BW, CEVA, Düsseldorf, Germany) was administered intramuscularly. After a sufficient depth of anaesthesia had been reached, animals were inoculated with 300 *A. abstrusus* L3 by injecting the inoculation dose directly into the stomach via a gastric tube. The gastric tube was flushed with tap water to ensure administration of the complete inoculation dose and was removed after flushing. Cats were observed for signs of regurgitation or vomiting at 10 (± 2) min and 1 h (± 10 min) after inoculation. None of the 27 cats vomited within this period, so no animals had to be re-inoculated, but all of the cats vomited between 1 h after inoculation and the next morning. One animal [ID 9713] of the control group G4 was prematurely excluded on SD 79 due to an anaesthesia incident during CT examination.

### Efficacy criteria

To promote the 3R principles, animals were not necropsied for efficacy evaluation, but faecal L1 counts were determined as primary efficacy criterion, and respiratory assessment, computed tomography (CT) and absence of anti-*A. abstrusus* MSP antibody development served as secondary efficacy criteria.

#### Faecal larvae counts

Between 30 to 46 dpi (SD 114 to 130), individual faecal samples were collected daily from each cat to determine excretion of *A. abstrusus* L1 via the Baermann technique (Fig. 1). Faecal samples of 5 g were either processed on the same day upon collection on weekdays or stored at 3.0–9.5 ˚C and processed on the following weekday upon collection on the weekend. L1 were allowed to migrate overnight, and larvae counts were performed the next morning.

#### Respiratory assessment

Clinical respiratory assessments of study animals were performed before inclusion in the study (SD −1), before infection (SD 83) and twice weekly after infection from 1 dpi (SD 85) until 50 dpi (SD 134) (Fig. 1). Examination for clinical respiratory signs involved the assessment of the respiratory frequency (breaths per minute), intensity of inspiratory and expiratory sounds (no, slight, moderate or severe sound), quality of respiratory sound (normal, deepened normal, stertor, stridor, rhonchus, wheeze, crackle), abdominal involvement (yes or no), panting (yes or no) and cough/retch (yes or no) as described previously [34].

#### Computed tomography (CT)

CT examinations of the cats were performed prior to study inclusion, before infection and once during the patency period. Because these examinations were time-intensive, several days were required to complete examinations of all study animals, i.e., SD −7 to −2, SD 75 to 78 and SD 131 to 134 (47 to 50 dpi) (Fig. 1). For anaesthesia, an intravenous catheter was placed in the cephalic vein, then the cats were premedicated with 0.3 mg/kg BW midazolame (MIDAzolam, B. Braun Melsungen AG, Melsungen, Germany) and 0.15 mg/kg BW levomethadone combined with 0.0075 mg/kg BW fenpipramide (L-Polamivet^®^, Intervet Deutschland GmbH, Unterschleißheim, Germany). Anaesthesia was induced by intravenous application of propofol (Narcofol^®^, CP-Pharma GmbH, Burgdorf, Germany) to effect. The cats were intubated and connected to a breathing circuit. Maintenance was performed with isoflurane (Isofluran CP^®^, CP-Pharma GmbH, Burgdorf, Germany) in 100% oxygen. End-tidal CO_2_ values were kept between 35 and 45 mmHg by mechanical ventilation.

Cats underwent CT examinations positioned in sternal recumbency, and scans were conducted using a 64-slice helical CT scanner (Philips Brilliance 64, Philips Healthcare, Amsterdam, the Netherlands). The image acquisition parameters were set as follows: 120 kVp, 150 mAs, rotation time of 0.5 s, and collimator pitch of 1. The scan field was individually optimized to encompass the entire lung field. A scan was performed during apnoea. Reconstruction parameters were configured with a slice thickness of 0.67 mm in a 512 × 512 matrix, an overlap of 0.33 mm, utilizing both a soft tissue algorithm (window level 60, window width 400) and a lung algorithm (window level −600, window width 1600). The images were transferred to a dedicated workstation (Philips Brilliance Workspace 3.0), and multiplanar reconstructions were reviewed according to the procedure described below.

All CT studies were assessed by a veterinarian holding a German Board specialist certification in diagnostic imaging. At the time of both image acquisition and assessment, the veterinarian was aware that the cats had been enrolled in a study evaluating the preventive efficacy against *A. abstrusus*. However, no information regarding the course of infection, clinical signs, or the date of treatment was disclosed. The CT lung findings for each cat were documented and categorized using a previously established system for assessing *A. abstrusus* images in cats [22]. The lungs were divided into three zones, with the first zone representing the 1-mm region at the periphery of each lung lobe, the second zone encompassing 5% of the lobar width beneath the visceral pleura, and the third zone covering the remaining lung parenchyma, including the peribronchovascular regions. For each zone, the presence of the following lung findings and patterns was documented: linear and reticular patterns, single nodules or a generalized nodular pattern, areas of high attenuation not attributable to nodules (such as ground-glass opacity, consolidation, marked or severe atelectasis), low attenuation (including air trapping, cystic lesions, bullae, bronchiectasis, emphysema) and a mosaic attenuation pattern (characterized by a patchwork of areas with different attenuation levels). The presence of parenchymal bands was also noted. Subjective evaluation was employed to assess bronchial wall thickness, which was described as either normal or thickened. Lastly, each lung was assigned a severity score for total CT changes, using a previously published scoring system as a reference [22], as illustrated in Additional file 1: Table S2.

#### Blood sampling and anti-*A. abstrusus* major sperm protein (MSP) antibody ELISA

Blood samples were collected once between SD −7 to SD −2, on SD 83 and once weekly starting in the week of 12 dpi (SD 96) until 78 dpi (SD 162) (Fig. 1). Individual blood samples were obtained by direct venipuncture of the right or left vena cephalica antebrachii, vena femoralis or vena saphena medialis. Blood samples were stored at 4 °C until centrifugation at 1500 × *g* for 10 min at room temperature for serum separation. Serum samples were stored at −20 °C until shipping to the Institute of Parasitology, Vetsuisse Faculty, University of Zurich, Switzerland, for enzyme-linked immunosorbent assay (ELISA) analysis to detect anti- *A. abstrusus* MSP antibodies. The ELISA was performed as described by Zottler et al. [28] and Schnyder et al. [41]. Briefly, Nunc Immobilizer Amino Plates (Thermo Fisher Scientific, Roskilde, Denmark) coated with recombinant *Dictyocaulus viviparus* MSP (0.25 µg/well), serum diluted 1:200, and a goat anti-feline IgG peroxidase-labelled conjugate (Southern Biotech, Birmingham, AL, USA) at a dilution of 1:9000 was used. The absorbance at a wavelength of 492 nm was read in a Multiscan ELISA reader (Tecan Infinite F50, Hombrechtikon, Switzerland). A conjugate control, substrate control, positive controls (sera from experimentally infected cats), negative controls (uninfected laboratory cats) and a reference served as plate correction factors for intra- and interspecific value adjustment. The cut-off was set to 0.194 optical density (OD) by calculating the mean OD values of the samples collected on 0 dpi and 14 dpi (SD 84 and 98) added up by three standard deviations.

### Statistical analysis and preventive efficacy

Statistical analysis was performed using the software package SAS^®^ (version 9.4; SAS Institute Inc., Cary, NC, USA).

The primary preventive efficacy criterion was the reduction of faecal larvae counts in each treatment group (G1-G3) in comparison with the control group (G4). Reduction of L1 counts was used to evaluate the percentage preventive efficacy in each treatment group according to the recommendations for controlled tests described in VICH GL7 [37] by the following formula:

$${\text{Efficacy reduction }}\left[ \% \right] \, = \frac{{\overline{X}_{C} - \overline{X}_{T} }}{{\overline{X}_{C} }} \times 100$$

where x̅_C_ is the GM number of individual maximum *A.* *abstrusus* faecal L1 counts in the control group (G4) and x̅_T_ is the GM number of individual maximum *A.* *abstrusus* L1 counts in each treatment group (G1–G3).

To allow the calculation in case of zero counts, the GM was calculated as follows:

$$x_{g} = \left( {\prod\limits_{i = 1}^{n} {(x_{i} + 1)} } \right)^{\frac{1}{n}} - 1$$

Individual maximum larval counts were log-transformed including a shift of 1 to allow transformation in case of zero counts: *x*’ = log_e_(*x* + 1). To test the adequacy of infection, the log-transformed individual maximum counts in the treatment groups were compared with the control group using a two-sided two-sample *t*-test.

The following parameters were considered as secondary preventive efficacy criteria:

(i) Respiratory parameters, including respiratory frequencies, intensity and quality of respiratory sound, panting, abdominal involvement and coughing or retching were evaluated but not analyzed statistically.
(ii) CT severity scores in the treatment groups were compared with the control group using two-sided Wilcoxon rank-sum test. Other CT findings (lung lobes affected, pleural changes, abnormalities in lung zones) were evaluated but not analyzed statistically.
(iii) Anti-*A. abstrusus* MSP antibody levels were evaluated but not analyzed statistically.

In all analyses, *p*-value ≤ 0.05 was considered statistically significant.

## Results

### Inclusion criteria and safety assessment

All 28 cats met the criteria for inclusion in the study. None of the animals showed abnormalities at the administration site after IVP application, and no adverse effects on general health were observed. Adverse events were reported for all 28 cats. There were mainly vomiting episodes during the wake-up phase after sedation and also some cases of *Giardia* infections, which were rated as non-serious and not related to treatment. Three serious adverse events occurred: two cats were diagnosed with hypertrophic cardiomyopathy, and one cat of the control group G4 experienced an anaesthesia incident during CT examination on SD 79; all were determined to be unrelated to treatment. The latter cat was withdrawn from the study.

### Faecal larvae counts

Faecal L1 counts were assessed from 30 to 46 dpi (SD 114 to 130). On 33 dpi (SD 117), the first cat of the control group (G4) started to excrete *A.* *abstrusus* L1, whereas on 37 dpi (SD 121) all cats of G4 had excreted L1 at least once and remained positive throughout the study period from day 41 to 46 dpi (SD 125 to 130). While only one animal each of G1 and G2 excreted larvae at two consecutive days (G1 on 38/39 dpi [SD 122/123] and G2 on 39/40 dpi [SD 123/124]), four cats of G1, two of G2 and three of G3 were positive on single occasions between 38 to 46 dpi (SD 122 to 130).

**Fig. 2:**
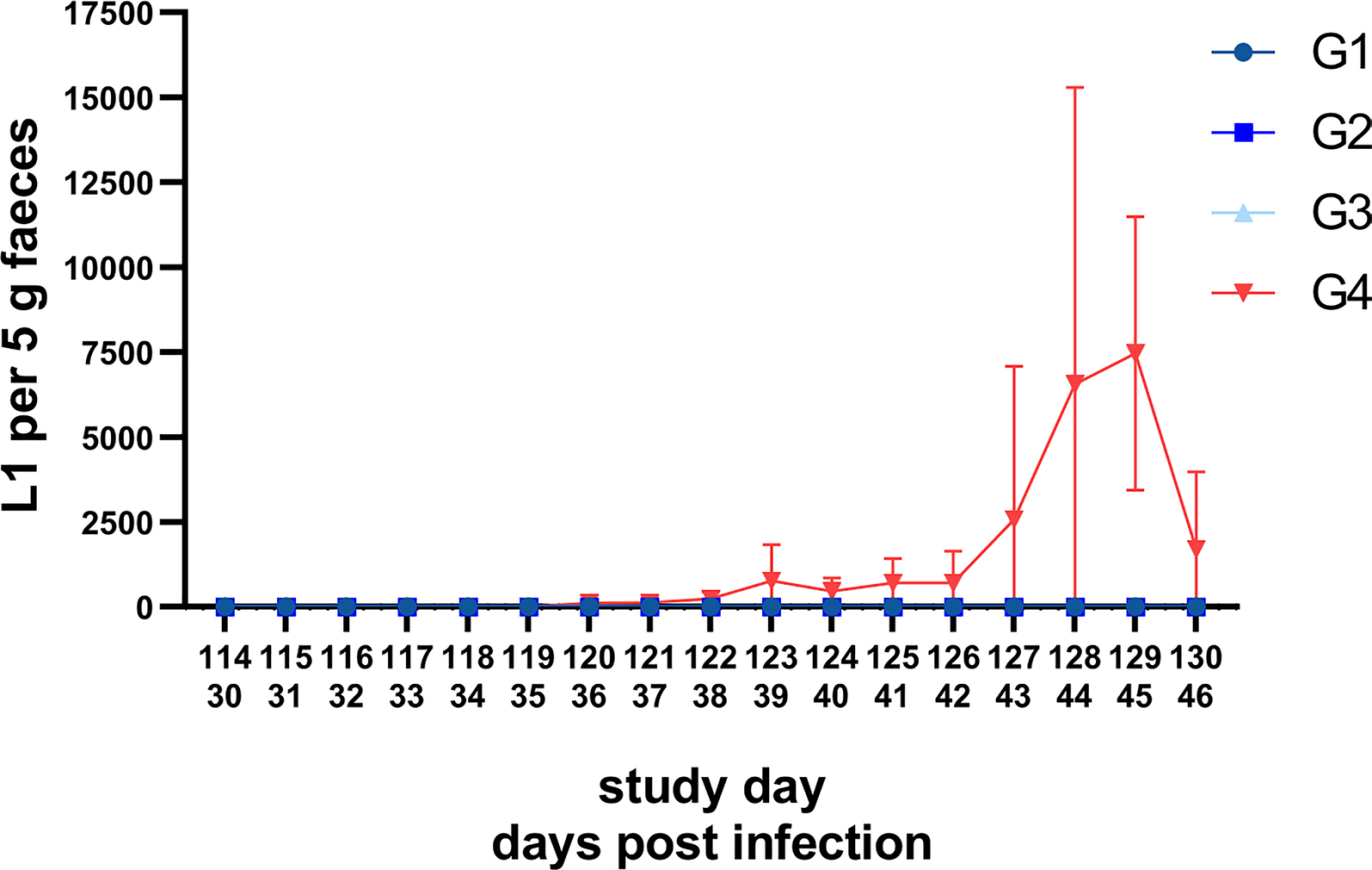
Faecal *A. abstrusus* first-stage larvae (L1) excretion of the investigational veterinary product (IVP)-treated groups (Bravecto^®^ Plus spot-on, minimum recommended dose of 40 mg fluralaner and 2.0 mg moxidectin/kg body weight; G1, G2, G3) and the control group (0.9% saline, G4). G1: treatment 12 weeks prior to inoculation; G2: treatment 8 weeks prior to inoculation; G3: treatment 4 weeks prior to inoculation

The maximum number of excreted L1 per 5 g of faeces per day varied between 2370 and 22,104 with a GM of 7380.89 in the control group (G4). In the treatment groups, low maximum larval counts of 1 to 8 with a GM of 1.63 in G1, 6 to 29 with a GM of 1.37 in G2, and 2 to 4 with a GM of 0.79 in G3 were observed. Requirements for adequacy of infection of the control group (G4) were met as shown by the significant differences of the log-transformed individual maximum larval counts of the control group (G4) of 8.91 versus those of the treatment groups, i.e. 0.97 for G1, 0.86 for G2 and 0.58 for G3 (two-sided *t*-test, *p* < 0.0001). Hence, the study was considered valid according to VICH GL7 [37] and VICH GL20 [42]. From 38 dpi (SD 122) onwards, larval excretion of the control group (G4) rose, whereas excretion stayed constantly low in the treatment groups (G1–G3) (Fig. 2).

Preventive efficacy of the IVP treatment against *A.* *abstrusus* L1 excretion was shown for every treatment group. A 99.99% reduction of GM maximum larval counts compared with those of the control group was shown upon IVP application 4 weeks prior to experimental infection (G3). When applied 8 (G2) or 12 (G1) weeks before infection, GM maximum larval counts were reduced by 99.89%. An overview on parasitological parameters for each animal and resulting efficacies is provided in Table 1.

**Table 1:** Parasitological parameters of the 27 study animals of the investigational veterinary product (IVP)-treated groups (Bravecto^®^ Plus spot-on solution, minimum recommended dose of 40 mg fluralaner and 2.0 mg moxidectin/kg body weight; G1, G2, G3) and the control group (0.9% saline; G4)

Group	Animal ID	First day of L1 excretion (dpi/SD)	Max. L1 count/day in 5 g faeces	GM of max. L1 count/day	Efficacy [%]	*p*-value
G1 (IVP treatment 12 weeks prior to infection)	1270	38/122^a^	2	1.63	99.98	<0.0001*
	2827	n.a.	0			
	3098	42/126^a^	1			
	3132	38/122^b^	1			
	3136	n.a.	0			
	3185	38/122^a^	8			
	3199	41/125^a^	7			
G2 (IVP treatment 8 weeks prior to infection)	0157	n.a.	0	1.37	99.98	<0.0001*
	1016	46/130^a^	1			
	1149	41/125^a^	29			
	1781	n.a.	0			
	2690	n.a.	0			
	2955	39/123^b^	6			
	3283	n.a.	0			
G3 (IVP treatment 4 weeks prior to infection)	0362	43/127^a^	2	0.79	99.99	<0.0001*
	2355	n.a.	0			
	2788	n.a.	0			
	3029	n.a.	0			
	3046	43/127^a^	4			
	3150	n.a.	0			
	3207	45/129^a^	3			
G4 (no IVP treatment, control)	0262	37/121	4170	7380.89	–	–
	1771	35/119	10140			
	2510	33/117	22104			
	2659	36/120	6171			
	2681	36/120	2370			
	2816	34/118	11825			

Preventive drug efficacy with comparison of log-transformed individual maximum first-stage larvae (L1) counts of the IVP treatment groups versus the control group by two-sided two-sample *t*-test. Note that one animal of the control group was excluded prior to infection on SD 79 due to an anaesthesia incident during CT examination

n.a.: No larvae excretion during the entire study

* Statistically significant by two-sided two-sample *t*-test

^a^ Larval excretion on single occasions

^b^ Larval excretion on two consecutive days

### Respiratory parameters

**Fig. 3:**
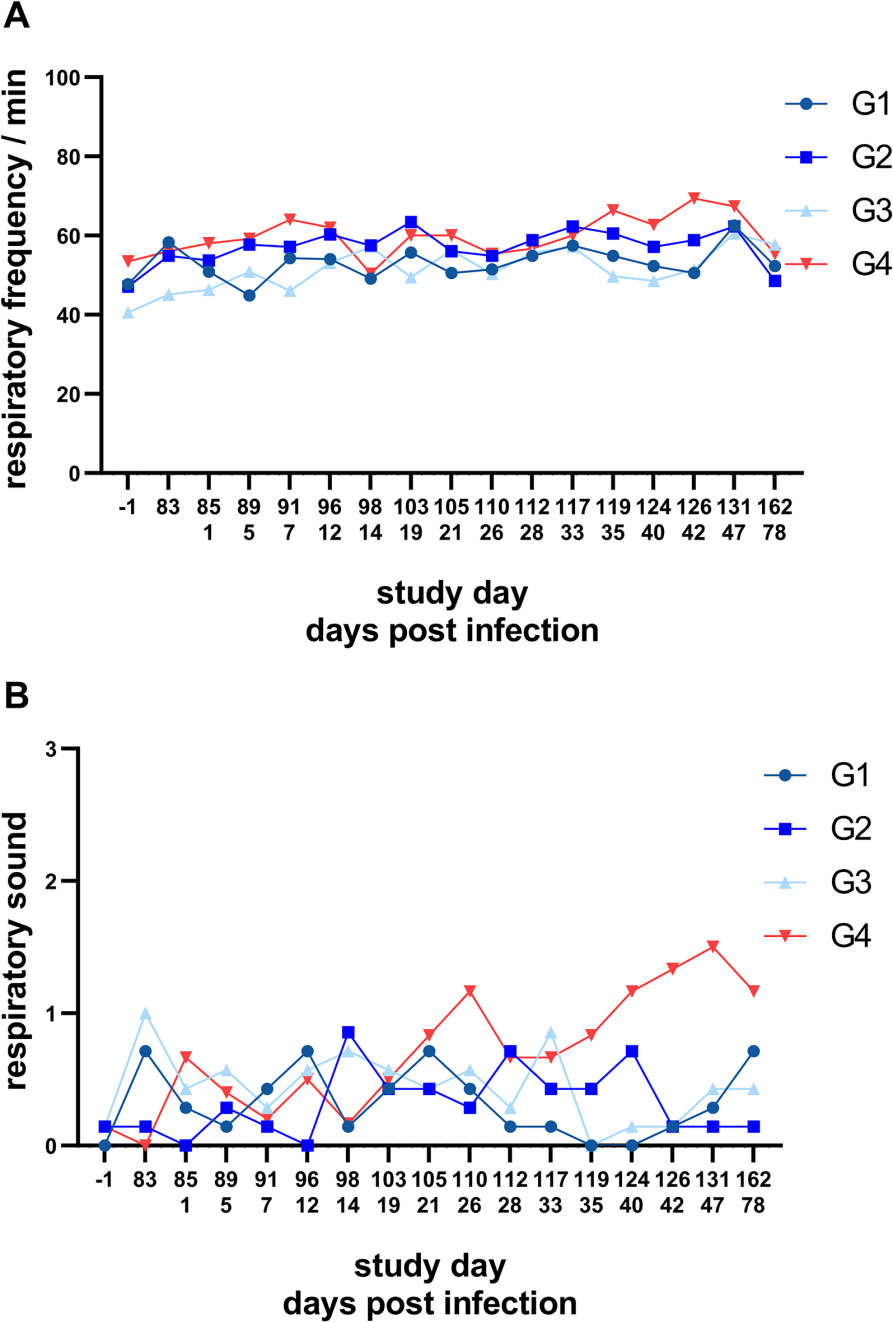
Respiratory frequencies (**A**) and intensities of respiratory sound (**B**) of the investigational veterinary product (IVP)-treated groups (Bravecto^®^ Plus spot-on, minimum recommended dose of 40 mg fluralaner and 2.0 mg moxidectin/kg body weight; G1, G2, G3) and the control group (0.9% saline, G4). Green arrows mark the anthelmintic treatment at the end of the study on 50 dpi (study day 134) and 64 dpi (study day 148). G1: treatment 12 weeks prior to inoculation; G2: treatment 8 weeks prior to inoculation; G3: treatment 4 weeks prior to inoculation

Both respiratory frequencies and intensity of respiratory sound fluctuated to a certain extent in all groups throughout the study. However, respiratory sound intensity of the control group G4 showed consistently higher values than those of the groups G1–G3 from 35 dpi (SD 119) onwards (Fig. 3A, B). No difference of the quality of respiratory sound was observed between the study groups throughout the course of the study. Moreover, none of the study cats showed cough/retch or panting. In three cats, abdominal involvement was observed, i.e. one at 14 dpi (SD 96) in G1, and one each at 26 dpi (SD 110) and 40 dpi (SD 124) in G4.

### Computed tomography (CT)

**Fig. 4:**
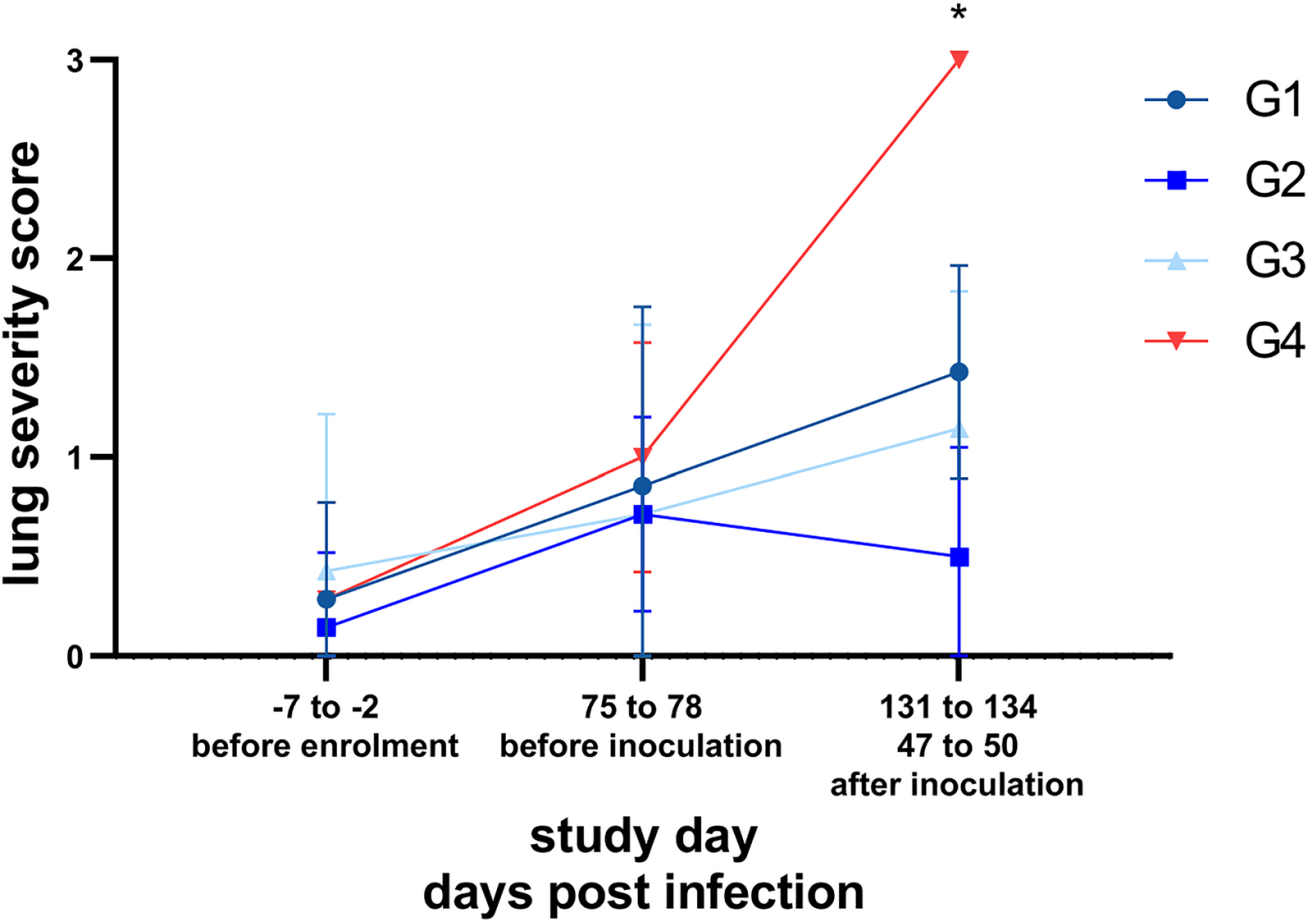
Lung severity scores of the investigational veterinary product (IVP)-treated groups (Bravecto^®^ Plus spot-on, minimum recommended dose of 40 mg fluralaner and 2.0 mg moxidectin/kg body weight; G1, G2, G3) and the control group (0.9% saline, G4). Comparison of lung severity scores of the treatment groups with the control group by two-sided Wilcoxon rank-sum test. G1: treatment 12 weeks prior to inoculation; G2: treatment 8 weeks prior to inoculation; G3: treatment 4 weeks prior to inoculation

The mean overall severity scores for all study groups at each time point are presented in Table 2. Prior to enrolment (SD −7 to SD −2), all cats in every group exhibited severity scores of 0 (none) or 1 (mild), except for one cat (ID 3207) in G3 showing a grade of 2 (moderate). No significant differences in severity scores were observed when comparing treatment groups with the control group G4 before enrolment. Following infection on 47 to 50 dpi (SD 131 to 134), all cats in the control group G4 were assigned a grade of 3, whereas cats in treatment groups showed significantly lower scores (Wilcoxon rank-sum test, G1 *p* = 0.0006; G2 *p* = 0.0022; G3 *p* = 0.0006) (Fig. 4).

**Table 2:** Mean lung severity scores of the investigational veterinary product (IVP)-treated groups (Bravecto^®^ Plus spot-on, minimum recommended dose of 40 mg fluralaner and 2.0 mg moxidectin/kg body weight; G1, G2, G3) and the control group (0.9% saline; G4). Comparison of mean lung severity scores of the IVP-treatment groups versus the control group by two-sided Wilcoxon rank-sum test

Group	Pre-enrolment (SD −7 to −2)		Before inoculation (SD 75 to 78)		Post inoculation (SD 131 to 134)	
	Mean	*p*-Value	Mean	*p*-Value	Mean	*p*-value
G1 (IVP treatment 12 weeks prior to infection)	0.29	1.0000	0.86	0.6166	1.43	0.0006*
G2 (IVP treatment 8 weeks prior to infection)	0.14	1.0000	0.71	0.6329	0.50	0.0022*
G3 (IVP treatment 4 weeks prior to infection)	0.43	1.0000	0.71	0.3502	1.14	0.0006*
G4 (no IVP treatment, control)	0.29	–	1.00	–	3.00	–

*Statistically significant by two-sided Wilcoxon rank-sum test

**Fig. 5:**
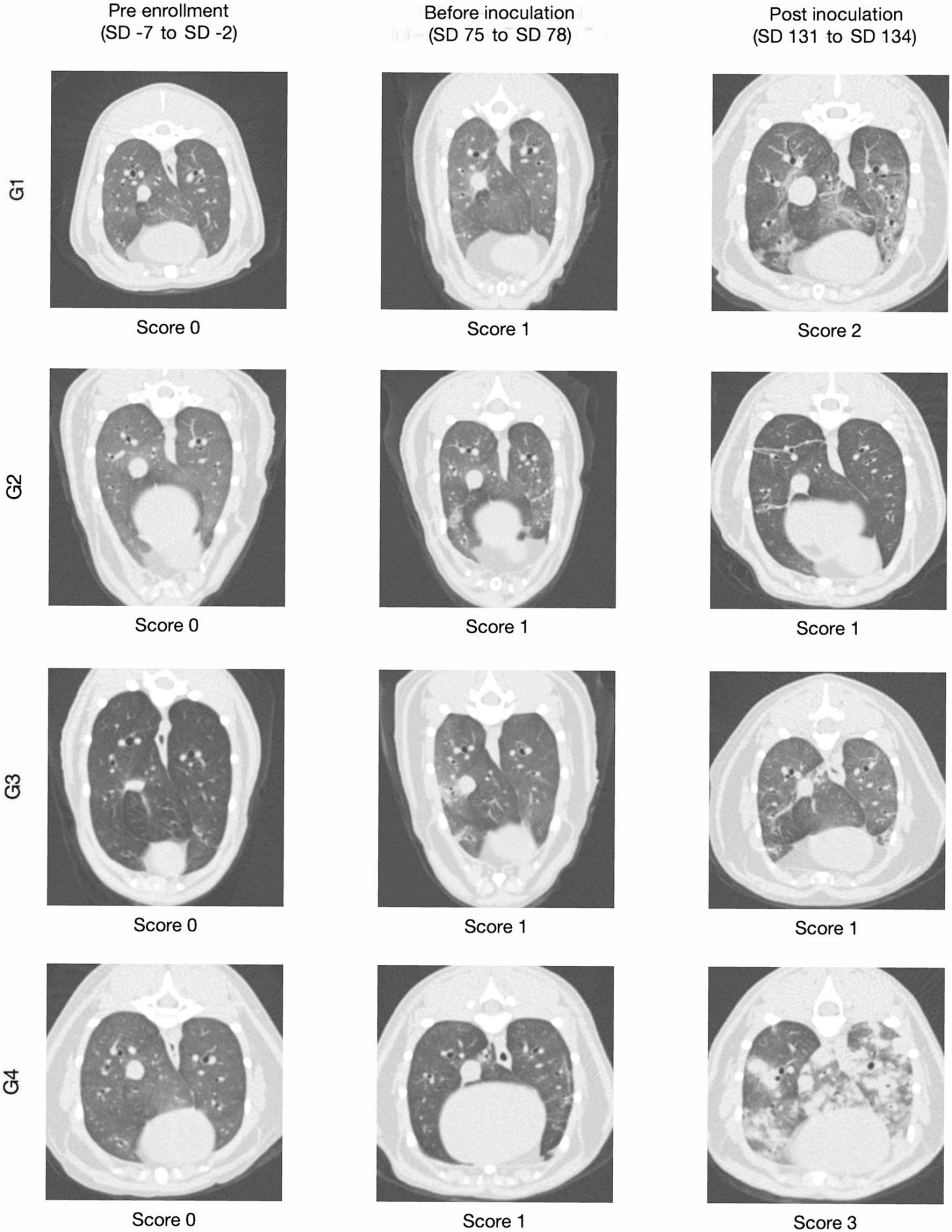
Representative transverse thoracic computed tomography (CT) images at the level of the caudal lung lobes of one cat in each of the investigational veterinary product (IVP)-treated groups (Bravecto^®^ Plus spot-on, minimum recommended dose of 40 mg fluralaner and 2.0 mg moxidectin/kg body weight; G1, G2, G3) and the control group (0.9% saline, G4). The given severity score is shown below each image. G1: treatment 12 weeks prior to inoculation; G2: treatment 8 weeks prior to inoculation; G3: treatment 4 weeks prior to inoculation

Most affected lungs displayed alterations in multiple lung lobes and bilaterally. When abnormalities were present, they were typically observed across all three lung zones. These abnormalities encompassed various patterns, such as areas of high attenuation (consolidation, atelectasis, ground glass opacification), linear and reticular changes, peripheral bands and mosaic-like patterns. However, nodules were exclusively identified in control group G4 at the end of the study (47 to 50 dpi, SD 131 to 134). Figure 5 illustrates representative transverse CT images of the thorax of a cat from each group at the three specified time points.

### Serology

**Fig. 6:**
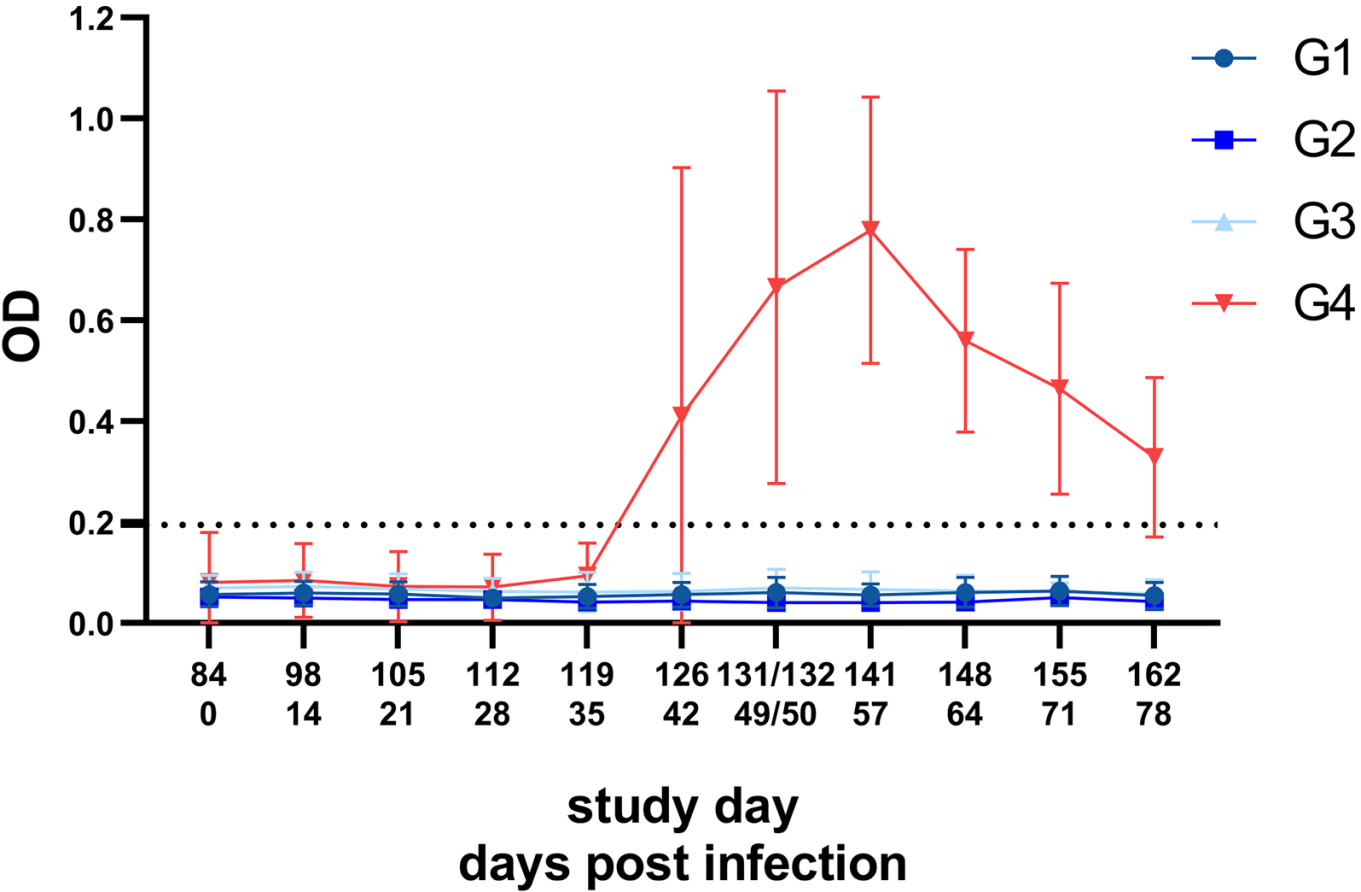
Anti-MSP *Aelurostrongylus abstrusus* antibody ELISA OD values of cats of the investigational veterinary product (IVP)-treated groups (Bravecto^®^ Plus spot-on, minimum recommended dose of 40 mg fluralaner and 2.0 mg moxidectin/kg body weight; G1, G2, G3) and the control group (0.9% saline, G4). Green arrows mark the anthelmintic treatment at the end of the study on 50 dpi (study day 134) and 64 dpi (study day 148). The dotted line indicates the cut-off value of 0.194 OD. G1: treatment 12 weeks prior to inoculation; G2: treatment 8 weeks prior to inoculation; G3: treatment 4 weeks prior to inoculation

OD values of the treatment groups G1, G2 and G3 stayed constantly low during the study period (Fig. 6). In contrast, OD values in the control group began to rise on 42 dpi (SD 126) until values reached a peak on 57 dpi (SD 141) (Fig. 6). Shortly after anthelmintic treatment on 50 dpi (SD 134) and 64 dpi (SD 148), OD values fell until the end of the study (78 dpi, SD 162).

## Discussion

According to the regulation (EU) 2019/6 of the European Parliament and of the Council [43] and the guideline VICH GL7 [37] adopted by the EMA, the efficacy of an anthelmintic agent is to be demonstrated by at least two controlled terminal dose confirmation studies. This study is the second controlled GCP study confirming the long-term preventive efficacy of at least 12 weeks of Bravecto^®^ Plus spot-on solution (280 mg/ml fluralaner and 14 mg/ml moxidectin) at the minimum recommended dose of 40 mg fluralaner and 2.0 mg moxidectin/kg BW against *A. abstrusus* in experimentally infected cat as shown by Raue et al. [34] in the first dose confirmation study.

In contrast to the first dose confirmation study, alternative non-terminal criteria, i.e. faecal larvae counts, respiratory parameters, CT findings and anti-*A. abstrusus* MSP antibody levels, were applied for the determination of efficacy by means of an innovative 3R-compliant concept as proposed by Raue et al. [22]. This approach is consistent with the animal welfare-related recommendation for evaluating the efficacy of anthelmintics against lungworms in dogs and cats issued by the World Association for the Advancement of Veterinary Parasitology (WAAVP), which recommends evaluation of other diagnostic methods and clinical parameters in addition to faecal larval excretion [44].

Here, the primary criterion for efficacy determination were *A. abstrusus* faecal L1 counts, which showed reductions of 99.98% after IVP application 12 or 8 weeks prior to experimental infection, and 99.99% after IVP application 4 weeks prior to experimental infection when compared with the control group. Hence, the efficacy was above the threshold of 90% as proposed by VICH GL7 [37], VICH GL20 [42] and required by EMA. While larval excretion was constantly high in the control group, only a total of 11 of the animals of the treatment groups excreted larvae, and that only sporadically, i.e. either on single occasions or two consecutive days. Moreover, excreted larvae numbers in the treatment groups were rather low, ranging from 1 to 29 L1, whereas up to 22,104 L1 were excreted by the animals of the control group, thus leading to an efficacy of almost 100% of Bravecto^®^ Plus spot-on solution against larval excretion upon challenge infection 4, 8 and 12 weeks after treatment, respectively. Nevertheless, larval excretion of some animals of the treatment groups indicated a patent infection of these animals, although the number of adults worms can be considered very low.

Whether the animals not showing larvae excretion harboured some adults remains unclear, as the lungs were not examined in this non-terminal study and accordingly no necropsies were performed. In this context, it was shown that larval excretion might be absent even though adults were present in lung tissue [19], and that the Baermann method for the detection of larvae may lose sensitivity upon intermittent or low excretion of larvae [19, 25]. Nevertheless, in the first dose confirmation study, similarly sporadic faecal larvae counts were also observed in the animals of the treatment groups, while in only one cat of the treatment groups an adult *A. abstrusus* could be retrieved from the lungs [34]. This may suggest very low worm numbers even in the animals that sporadically excreted larvae in the present study.

To supplement the assessment of faecal larvae counts with additional data, further secondary efficacy criteria were implemented as additional target parameters. One of these parameters was the detection of anti-*A. abstrusus* MSP antibodies. Antibody levels correlated with larval excretion as shown by seroconversion of the control animals around the beginning of patency and the constantly low OD levels in the treatment groups. Moreover, OD values substantially fell in the control group shortly after anthelmintic treatment on 50 and 64 dpi (SD 134 and SD 148), which is in concordance with literature reporting the short persistence of anti-*A. abstrusus* MSP antibodies [22, 28, 45]. As the ELISA utilizes bovine lungworm MSP as diagnostic antigen [27], which is abundant in sperm of adult males [46], high L1 excretion might be linked to the presence of many adult *A. abstrusus* males as previously proposed by Raue et al. [22]. Interestingly, even though some animals of the treatment groups occasionally excreted larvae, seroconversion was not observed, indicating that antibodies were absent due to very low numbers of adult males or below the detection limit. In this context, a sensitivity of 100% and 88.2% for sera of experimentally and naturally infected cats, respectively, was shown by Zottler et al. [28] and similar findings were observed by Raue et al. [22] in a cat excreting low numbers of L1, namely 0.5 L1 per gram of faeces. Interestingly, the lung of this cat presented changes attributable to a lungworm infection in CT, showing the value of supplementing parasitological and serological parameters by imaging methods to detect subclinical infections [23, 47].

In the present study, animals of the control group showed marked pulmonary changes in CT after infection as indicated by a lung severity score of 3 in all control cats. These abnormalities are consistent with previous findings [22–24], showing areas of high attenuation (consolidation, atelectasis, ground-glass opacification), linear and reticular changes, peripheral bands, mosaic-like patterns and nodules. While nodules were exclusively found in the control group after infection, other abnormalities, i.e. mostly high attenuation, linear and reticular changes and rarely mosaic-like patterns, were also observed in the cats of the treatment groups. Nevertheless, only the pulmonary changes in the control group after infection were highly indicative of a lungworm infection, since cats of both the treatment and control groups showed slight changes already before enrolment and experimental infection. Moreover, only the lung severity scores of the control group animals considerably increased from a mean of 1.0 shortly before infection to a mean of 3.0 after infection, whereas those of the treatment groups either rose only slightly (G1, G3) or even declined (G2). Possible reasons for the abnormalities before experimental infection, some of which could have been positioning- or narcosis-related atelectasis [48, 49] or previous lung disease, are debatable. The animals were obtained from the same breeder as in the first dose confirmation study, where cats showed signs of an outbreak of a mild, apyretic upper respiratory disease (cat flu) with similar events already observed in the breeding facility [22]. However, in the present study, none of the cats showed clinical signs of this or any other respiratory disease. Hence, pulmonary changes might rather be related to atelectasis due to narcosis or positioning of cats for CT examinations [48, 49].

While faecal larvae counts, serological and imaging data corresponded well, respiratory parameters were less conclusive. In general, respiratory frequencies and respiratory sound intensity varied between examination days and individual cats throughout the study, which is not surprising considering the fact that, for instance, excitement of the cats during examination or purring might have had an influence on respiratory parameters [19, 22]. Interestingly, the respiratory sound intensity values of the control group were consistently above those of the treatment groups as of the onset of patency. However, previous studies reported either no relation of both respiratory frequency and sound intensity to *A. abstrusus* infection or only by trend [19, 22, 34]. Therefore, these parameters may only be suitable as additional criteria and not as secondary efficacy criteria in efficacy studies.

Containing both fluralaner and moxidectin, Bravecto^®^ Plus is a broad-spectrum endectoparasiticide that can be used for the treatment and/or prevention of a variety of different parasites. This is especially valuable for cats with outdoor access being at high risk to acquire endo- and ectoparasites simultaneously. Multiparasitism in cats is commonly detected all over the world [13, 50–53]. For instance, Beugnet et al. [51] showed in a European-wide study spanning Austria, Belgium, France, Hungary, Italy, Romania and Spain that 14.0% of the 1519 examined owned domestic cats were coinfected/coinfested with endo- and ectoparasites, highlighting the need for effective and regular control measures [50]. A prerequisite for this is sufficient cat owner compliance, which can be improved by a reduced frequency of treatments and as few products as possible to be used at each time of treatment. Accordingly, Bravecto^®^ Plus holds the potential for an improved owner compliance as administration in 12-week intervals protects cats from common ecto- and endoparasitic diseases [34, 54–57].

## Conclusion

﻿In a single administration of Bravecto^®^ Plus spot-on solution for cats (280 mg/ml fluralaner and 14 mg/ml moxidectin) was well tolerated and effective in the prevention of cat aelurostrongylosis for at least 12 weeks by limiting the establishment of adult *A. abstrusus* in the lungs and preventing associated lung damage and larval excretion. Moreover, the multi-diagnostic approach incorporating faecal larvae counts, antibody levels, CT examinations and respiratory parameters allowed the assessment of the IVP efficacy for the prevention of aelurostrongylosis with a similar degree of certainty and accuracy as a parasitological necropsy, and therefore represents a contemporary and desirable alternative to the guideline-compliant, terminal methods of efficacy testing of anthelmintics. Nonetheless, a combination of terminal and non-terminal studies is still required by the EMA to prove adequate IVP efficacy. Therefore, future studies incorporating the proposed multi-diagnostic approach might aid in further defining thresholds and recommendations for discrimination of alternative non-terminal criteria, thus holding the potential for the complete replacement of terminal studies in the efficacy determination of anthelmintics in the future.

### Supplementary Information

**Additional file 1: Table S1.** Homogeneity of the four study groups. Distribution of age, weight and sex of the 27 study animals of the investigational veterinary product (IVP)-treated groups (Bravecto^®^ Plus spot-on, 2.0 mg moxidectin and 40 mg fluralaner/kg BW; G1, G2, G3) and the control group (0.9% saline, G4). Of the seven control animals, one was excluded on SD 79 due to an anaesthesia incident during CT examination. **Table S2.** Scoring system for the determination of severity of total changes in the lungs of cats in computed tomography (CT).

## Data Availability

Most data analysed during this study are included in the article. The remaining data from this clinical study are proprietary and maintained by MSD Animal Health.

## References

[1] Morelli S, Diakou A, Colombo M, Di Cesare A, Barlaam A, Dimzas D (2021). Cat respiratory nematodes: current knowledge, novel data and warranted studies on clinical features, treatment and control. Pathogens.

[2] Traversa D, Morelli S, Di Cesare A, Diakou A (2021). Felid cardiopulmonary nematodes: dilemmas solved and new questions posed. Pathogens.

[3] Giannelli A, Capelli G, Joachim A, Hinney B, Losson B, Kirkova Z (2017). Lungworms and gastrointestinal parasites of domestic cats: a European perspective. Int J Parasitol.

[4] Bourgoin G, Callait-Cardinal MP, Bouhsira E, Polack B, Bourdeau P, Roussel Ariza C (2022). Prevalence of major digestive and respiratory helminths in dogs and cats in France: results of a multicenter study. Parasit Vectors.

[5] Elsheikha HM, Wright I, Wang B, Schaper R (2019). Prevalence of feline lungworm *Aelurostrongylus abstrusus* in England. Vet Parasitol Reg Stud Rep.

[6] Garcia-Campos A, Power C, O'Shaughnessy J, Browne C, Lawlor A, McCarthy G (2019). One-year parasitological screening of stray dogs and cats in County Dublin. Ireland Parasitology.

[7] Grandi G, Comin A, Ibrahim O, Schaper R, Forshell U, Lind EO (2017). Prevalence of helminth and coccidian parasites in Swedish outdoor cats and the first report of *Aelurostrongylus abstrusus* in Sweden: a coprological investigation. Acta Vet Scand.

[8] Hansen AP, Skarbye LK, Vinther LM, Willesen JL, Pipper CB, Olsen CS (2017). Occurrence and clinical significance of *Aelurostrongylus abstrusus* and other endoparasites in Danish cats. Vet Parasitol.

[9] Henry P, Huck-Gendre C, Franc M, Williams TL, Bouhsira E, Lienard E (2022). Epidemiological survey on gastrointestinal and pulmonary parasites in cats around Toulouse (France). Helminthologia.

[10] Olsen CS, Willesen JL, Pipper CB, Mejer H (2015). Occurrence of *Aelurostrongylus abstrusus* (Railliet, 1898) in Danish cats: a modified lung digestion method for isolating adult worms. Vet Parasitol.

[11] Raue K, Heuer L, Bohm C, Wolken S, Epe C, Strube C (2017). 10-year parasitological examination results (2003 to 2012) of faecal samples from horses, ruminants, pigs, dogs, cats, rabbits and hedgehogs. Parasitol Res.

[12] Zottler EM, Bieri M, Basso W, Schnyder M (2019). Intestinal parasites and lungworms in stray, shelter and privately owned cats of Switzerland. Parasitol Int.

[13] Genchi M, Vismarra A, Zanet S, Morelli S, Galuppi R, Cringoli G (2021). Prevalence and risk factors associated with cat parasites in Italy: a multicenter study. Parasit Vectors.

[14] Kiszely S, Gyurkovszky M, Solymosi N, Farkas R (2019). Survey of lungworm infection of domestic cats in Hungary. Acta Vet Hung.

[15] Morelli S, Diakou A, Di Cesare A, Schnyder M, Colombo M, Strube C (2020). Feline lungworms in Greece: copromicroscopic, molecular and serological study. Parasitol Res.

[16] Remesar S, Garcia-Dios D, Calabuig N, Prieto A, Diaz-Cao JM, Lopez-Lorenzo G (2022). Cardiorespiratory nematodes and co-infections with gastrointestinal parasites in new arrivals at dog and cat shelters in north-western Spain. Transbound Emerg Dis.

[17] Crisi PE, Aste G, Traversa D, Di Cesare A, Febo E, Vignoli M (2017). Single and mixed feline lungworm infections: clinical, radiographic and therapeutic features of 26 cases (2013–2015). J Feline Med Surg.

[18] Philbey AW, Krause S, Jefferies R (2014). Verminous pneumonia and enteritis due to hyperinfection with *Aelurostrongylus abstrusus* in a kitten. J Comp Pathol.

[19] Schnyder M, Di Cesare A, Basso W, Guscetti F, Riond B, Glaus T (2014). Clinical, laboratory and pathological findings in cats experimentally infected with *Aelurostrongylus abstrusus*. Parasitol Res.

[20] Soares C, Cardoso M, Mestre A, Crisi PE (2017). Case report: severe and progressive bronchopneumonia by *Aelurostrongylus abstrusus* in an adopted stray cat from Portugal. J Parasit Dis.

[21] Hamilton JM (1970). The influence of infestation by *Aelurostrongylus abstrusus* on the pulmonary vasculature of the cat. Br Vet J.

[22] Raue K, Raue J, Hauck D, Sobbeler F, Morelli S, Traversa D (2021). Do all roads lead to Rome? The potential of different approaches to diagnose *Aelurostrongylus abstrusus* infection in cats. Pathogens.

[23] Dennler M, Bass DA, Gutierrez-Crespo B, Schnyder M, Guscetti F, Di Cesare A (2013). Thoracic computed tomography, angiographic computed tomography, and pathology findings in six cats experimentally infected with *Aelurostrongylus abstrusus*. Vet Radiol Ultrasound.

[24] Lacava G, Zini E, Marchesotti F, Domenech O, Romano F, Manzocchi S (2017). Computed tomography, radiology and echocardiography in cats naturally infected with *Aelurostrongylus abstrusus*. J Feline Med Surg.

[25] Traversa D, Di Cesare A (2016). Diagnosis and management of lungworm infections in cats: cornerstones, dilemmas and new avenues. J Feline Med Surg.

[26] Traversa D, Iorio R, Otranto D (2008). Diagnostic and clinical implications of a nested PCR specific for ribosomal DNA of the feline lungworm *Aelurostrongylus abstrusus* (Nematoda, Strongylida). J Clin Microbiol.

[27] von Holtum C, Strube C, Schnieder T, von Samson-Himmelstjerna G (2008). Development and evaluation of a recombinant antigen-based ELISA for serodiagnosis of cattle lungworm. Vet Parasitol.

[28] Zottler EM, Strube C, Schnyder M (2017). Detection of specific antibodies in cats infected with the lung nematode *Aelurostrongylus abstrusus*. Vet Parasitol.

[29] Böhm C, Wolken S, Schnyder M, Basso W, Deplazes P, Di Cesare A (2015). Efficacy of emodepside/praziquantel Spot-on (Profender(R)) against adult *Aelurostrongylus abstrusus* nematodes in experimentally infected cats. Parasitol Res.

[30] Cvejic D, Mencke N, Petry G, Ringeisen H, Hamburg H, Hellmann K (2022). Multicenter randomized, and blinded European field study evaluating the efficacy and safety of Felpreva^(R)^, a novel spot-on formulation containing tigolaner, emodepside and praziquantel, in treating cats with mixed infection with intestinal nematodes, cestodes and/or lungworms. Curr Res Parasitol Vector Borne Dis.

[31] Giannelli A, Brianti E, Varcasia A, Colella V, Tamponi C, Di Paola G (2015). Efficacy of Broadline^(R)^ spot-on against *Aelurostrongylus abstrusus* and *Troglostrongylus brevior* lungworms in naturally infected cats from Italy. Vet Parasitol.

[32] Knaus M, Chester ST, Rosentel J, Kuhnert A, Rehbein S (2014). Efficacy of a novel topical combination of fipronil, (S)-methoprene, eprinomectin and praziquantel against larval and adult stages of the cat lungworm, *Aelurostrongylus abstrusus*. Vet Parasitol.

[33] Heuer L, Petry G, Pollmeier M, Schaper R, Deuster K, Schmidt H (2020). Efficacy of imidacloprid 10%/moxidectin 1% spot-on formulation (Advocate^(R)^) in the prevention and treatment of feline aelurostrongylosis. Parasit Vectors.

[34] Raue K, Rohdich N, Hauck D, Zschiesche E, Morelli S, Traversa D (2021). Efficacy of Bravecto^(R)^ Plus spot-on solution for cats (280 mg/ml fluralaner and 14 mg/ml moxidectin) for the prevention of aelurostrongylosis in experimentally infected cats. Parasit Vectors.

[35] Traversa D, Joachim A (2018). The 3Rs concept: time to change how we evaluate the efficacy of anthelmintics in companion animals. Trends Parasitol.

[36] VICH. 9: Good clinical practice. Veterinary International Cooperation on Harmonization, European Agency for the Evaluation of Medicinal Products, London, CVMP/VICH/595/98-Final. 2000.

[37] VICH. 7: Efficacy of anthelmintics: general requirements - revision 1. Veterinary International Cooperation on Harmonization, European Agency for the Evaluation of Medicinal Products, London, EMA/CVMP/VICH/832/1999. 2022.

[38] Di Cesare A, Crisi PE, Di Giulio E, Veronesi F, Frangipane di Regalbono A, Talone T, et al. Larval development of the feline lungworm *Aelurostrongylus abstrusus* in *Helix aspersa*. Parasitol Res. 2013;112:3101–8. 10.1007/s00436-013-3484-2 23743614

[39] Di Cesare A, Veronesi F, Frangipane di Regalbono A, Iorio R, Traversa D. Novel molecular assay for simultaneous identification of neglected lungworms and heartworms affecting cats. J Clin Microbiol. 2015;53:3009–13. 10.1128/JCM.00901-15 PMC4540927 26109447

[40] Morelli S, Traversa D, Colombo M, Raue K, Strube C, Pollmeier M (2020). The effect of the hibernation on the larval development of *Troglostrongylus brevior* in the land snail *Cornu aspersum*. Vet Parasitol.

[41] Schnyder M, Schaper R, Gori F, Hafner C, Strube C (2021). *Aelurostrongylus abstrusus* antibody seroprevalence reveals that cats are at risk of infection throughout Germany. Pathogens.

[42] VICH. 20: Efficacy of anthelmintics: specific recommendations for felines - revision 1. Veterinary International Cooperation on Harmonization, European Agency for the Evaluation of Medicinal Products, London, EMA/CVMP/VICH/545/2000. 2022.

[43] Parliament E, Union tCotE. Regulation (EU) 2019/6 of the European Parliament and of the Council of 11 December 2018 on veterinary medicinal products and repealing Directive 2001/82/EC. Official J Eur Union. 2019;276:43-167.

[44] Beugnet F, Taweethavonsawat P, Traversa D, Fourie J, McCall J, Tielemans E (2022). World Association for the Advancement of Veterinary Parasitology (WAAVP): second edition of guidelines for evaluating the efficacy of anthelmintics for dogs and cats. Vet Parasitol..

[45] Di Cesare A, Gueldner EK, Traversa D, Veronesi F, Morelli S, Crisi PE (2019). Seroprevalence of antibodies against the cat lungworm *Aelurostrongylus abstrusus* in cats from endemic areas of Italy. Vet Parasitol.

[46] Strube C, Buschbaum S, Schnieder T (2009). Molecular characterization and real-time PCR transcriptional analysis of *Dictyocaulus viviparus* major sperm proteins. Parasitol Res.

[47] Febo E, Crisi PE, Traversa D, Luciani A, Di Tommaso M, Pantaleo S (2019). Comparison of clinical and imaging findings in cats with single and mixed lungworm infection. J Feline Med Surg.

[48] Foo TS, Pilton JL, Hall EJ, Martinez-Taboada F, Makara M (2018). Effect of body position and time on quantitative computed tomographic measurements of lung volume and attenuation in healthy anesthetized cats. Am J Vet Res.

[49] Oliveira CR, Mitchell MA, O'Brien RT (2011). Thoracic computed tomography in feline patients without use of chemical restraint. Vet Radiol Ultrasound.

[50] Beugnet F (2021). NexGard^(R)^ Combo (esafoxolaner, eprinomectin, praziquantel), a new endectoparasiticide spot-on formulation for cats. Parasite.

[51] Beugnet F, Bourdeau P, Chalvet-Monfray K, Cozma V, Farkas R, Guillot J (2014). Parasites of domestic owned cats in Europe: co-infestations and risk factors. Parasit Vectors.

[52] Colella V, Nguyen VL, Tan DY, Lu N, Fang F, Zhijuan Y (2020). Zoonotic vectorborne pathogens and ectoparasites of dogs and cats in Eastern and Southeast Asia. Emerg Infect Dis.

[53] Nagamori Y, Payton ME, Looper E, Apple H, Johnson EM (2020). Retrospective survey of parasitism identified in feces of client-owned cats in North America from 2007 through 2018. Vet Parasitol.

[54] Di Cesare A, Morelli S, Rohdich N, Kirkova Z, Capari B, Loehlein W (2024). Efficacy of a spot-on formulation containing 280 mg/ml fluralaner and 14 mg/ml moxidectin (Bravecto^(R)^ Plus) in the prevention of cat aelurostrongylosis under field conditions. Vet Parasitol.

[55] Rohdich N, Zschiesche E, Wolf O, Loehlein W, Kirkova Z, Iliev P (2018). A randomized, blinded, controlled, multi-centered field study assessing the treatment of gastrointestinal nematode infections in cats with fluralaner plus moxidectin spot-on solution (Bravecto^(R)^ Plus). Parasit Vectors.

[56] Rohdich N, Zschiesche E, Wolf O, Loehlein W, Pobel T, Gil MJ (2018). Field effectiveness and safety of fluralaner plus moxidectin (Bravecto^(R)^ Plus) against ticks and fleas: a European randomized, blinded, multicenter field study in naturally-infested client-owned cats. Parasit Vectors.

[57] Taenzler J, de Vos C, Roepke RKA, Heckeroth AR (2018). Efficacy of fluralaner plus moxidectin (Bravecto^(R)^ Plus spot-on solution for cats) against *Otodectes cynotis* infestations in cats. Parasit Vectors.

