# Supplementary material for: Efficacy of Bravecto® Plus spot-on solution for cats (280 mg/ml fluralaner and 14 mg/ml moxidectin) in the prevention of feline Aelurostrongylus abstrusus infection evaluated in a multi-diagnostic approach
Source: Parasit Vectors. 2024 Apr 24;17:193. doi: 10.1186/s13071-024-06270-0 (PMC11044604; doi:10.1186/s13071-024-06270-0)
Supplement: Supplementary file 1 — Additional file 1: Table S1. Homogeneity of the four study groups. Distribution of age, weight and sex of the 27 study animals of the investigational veterinary product (IVP)-treated groups (Bravecto® Plus spot-on, 2.0 mg moxidectin and 40 mg fluralaner/kg BW; G1, G2, G3) and the control group (0.9% saline, G4). Of the seven control animals, one was excluded on SD 79 due to an anaesthesia incident during CT examination. Table S2. Scoring system for the determination of severity of total changes in the lungs of cats in computed tomography (CT). [file 13071_2024_6270_MOESM1_ESM.docx]

**Supplementary Table 1:** Homogeneity of the four study groups. Distribution of age, weight and sex of the 27 study animals of the investigational veterinary product (IVP)-treated groups (Bravecto® Plus spot-on, 2.0 mg moxidectin and 40 mg fluralaner/kg BW; G1, G2, G3) and the control group (0.9% saline, G4). Of the seven control animals, one was excluded on SD 79 due to an anaesthesia incident during CT examination.

|  | Study group | IVP treatment | No. of cats | Mean | Std | Min | Median | Max | female/male |
| --- | --- | --- | --- | --- | --- | --- | --- | --- | --- |
| Age [months] | G1 | SD 0 | 7 | 6.9 | 0.4 | 6.0 | 7.0 | 7.0 | 3/4 |
|  | G2 | SD 28 | 7 | 6.9 | 0.4 | 6.0 | 7.0 | 7.0 | 2/5 |
|  | G3 | SD 56 | 7 | 6.7 | 0.5 | 6.0 | 7.0 | 7.0 | 3/4 |
|  | G4 | - | 7 | 6.7 | 0.5 | 6.0 | 7.0 | 7.0 | 2/5 |
|  | Total | | 28 | 6.8 | 0.4 | 6.0 | 7.0 | 7.0 | 10/18 |
| Weight [kg] | G1 | SD 0 | 7 | 3.2 | 0.6 | 2.2 | 3.4 | 3.9 | 3/4 |
|  | G2 | SD 28 | 7 | 3.6 | 0.5 | 2.9 | 3.8 | 4.4 | 2/5 |
|  | G3 | SD 56 | 7 | 3.3 | 0.6 | 2.6 | 3.3 | 4.0 | 3/4 |
|  | G4 | - | 7 | 3.6 | 0.4 | 2.9 | 3.6 | 4.2 | 2/5 |
|  | Total | | 28 | 3.4 | 0.5 | 2.2 | 365 | 4.4 | 10/18 |

**Supplementary Table 2:** Scoring system for the determination of severity of total changes in the lungs of cats in computed tomography (CT).

| Severity score | Definition |
| --- | --- |
| 0 none | No changes. |
| 1 mild | Some or all zones affected, with predominantly ground glass opacity with only occasional areas of consolidation. |
| 2 moderate | All zones affected, with multifocal areas of mixed attenuation (ground glass opacity and mosaic attenuation), change affecting multiple or all lobes. Occasional areas of consolidation. |
| 3 severe | Multiple areas to diffuse changes in all zones with clear areas of marked hyperattenuation and consolidation resulting in loss of vascular margins. Accompanied by marked ground glass opacity. Maybe coexisting features of bronchiectasis or air-trapping resulting in mosaic attenuation pattern. |
